# Controlling Ion Conductance and Channels to Achieve Synaptic-like Frequency Selectivity

**DOI:** 10.1007/s40820-014-0024-2

**Published:** 2014-12-16

**Authors:** Siheng Lu, Fei Zeng, Wenshuai Dong, Ao Liu, Xiaojun Li, Jingting Luo

**Affiliations:** 1 Laboratory of Advanced Materials (MOE), School of Materials Science and Engineering, Tsinghua University, Beijing, People’s Republic of China; 2 Institute of Thin Film Physics and Applications, Shenzhen Key Laboratory of Sensor Technology, Shenzhen University, Shenzhen, People’s Republic of China

**Keywords:** Ions migration, Nano-channels, Frequency selectivity, Semiconducting polymer, Organic electrolyte, Dynamic doping

## Abstract

Enhancing ion conductance and controlling transport pathway in organic electrolyte could be used to modulate ionic kinetics to handle signals. In a Pt/Poly(3-hexylthiophene-2,5-diyl)/Polyethylene+LiCF_3_SO_3_/Pt hetero-junction, the electrolyte layer handled at high temperature showed nano-fiber microstructures accompanied with greatly improved salt solubility. Ions with high mobility were confined in the nano-fibrous channels leading to the semiconducting polymer layer, which is favorable for modulating dynamic doping at the semiconducting polymer/electrolyte interface by pulse frequency. Such a device realized synaptic-like frequency selectivity, i.e., depression at low frequency stimulation but potentiation at high-frequency stimulation.

## Introduction

 Ion transportation in organic electrolyte has been widely studied, especially in the field of advanced energy [1]. State of electrolyte and ion conductance influenced significantly performance of ion battery [2]. Early study indicated that ion conductivity had contributions from both anions and cations when the electrolyte was amorphous [3]. Since working voltage is usually high in ion battery, ion transport couples strongly with polymer segments in kinetics and then high ionic mobility along fixed direction is required to weaken this coupling [4, 5]. Thus, crystalline polymer electrolyte was proposed and fabricated to enhance ionic conductivity, in which ions could move in designed channels quickly [6]. Some study demonstrates the ionic pathway control by 3D network design [7].

In this paper, we focused on how electrolyte-based device behaves under small input in a situation of directional ionic channels formed, and how that can be related to information handling. We have found a synaptic-like frequency selectivity, a frequency dependent response transformed between depression to potentiation, of Pt/Poly(3-hexylthiophene-2,5-diyl) (P3HT)/Polyethylene (PEO)+LiCF_3_SO_3_/Pt cell in previous study [8]. This device could be applicable to signal handling and synaptic computation, such as filtering and decoding. Compared with those memristors simulating synaptic plasticity, which is a popular research topic in the fields of informatics, materials, and neuroscience [9–13], our cell owns several advantages. For example, ions are working substances, dynamic doping is reversible, and power consumption is low. Therefore, this type of device has great potential to be artificial synapse for brain-like computation [14]. We further studied microstructure of this device in this paper and found that ions migration along directional channels was a necessary factor to obtain frequency selectivity.

## Experimental

**Fig. 1:**
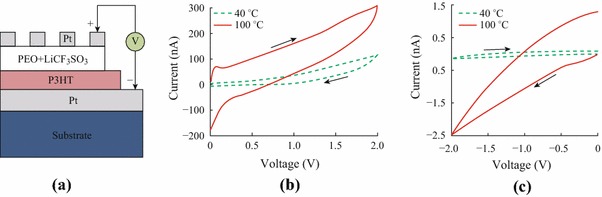
**a** Schematic of the Pt/P3HT/PEO + LiCF_3_SO_3_/Pt cell. Current–voltage (*I*–*V*) curves: where the sweeping direction was **b** 0 → 2→0 V and; **c** 0 → −2 → 0 V; and the sweep rate was 100 V s^−1^

Substrates (1.2 × 1.2 cm) were cut from the commercially available Si/SiO_2_(300 nm)/Ti(20 nm)/Pt(150 nm) wafers, in which Pt (150 nm) was used as the bottom electrode (BE). The substrates were cleaned using ultrasonic waves in a sequence of acetone, ethyl alcohol, deionized water, and ethyl alcohol, then dried using a stream of nitrogen gas and stored in a nitrogen atmosphere glove box. Poly(3-hexylthiophene-2,5-diyl) (P3HT) was purchased from Zhejiang Optical & Electronic Technology Co. Ltd. Polyethylene (PEO) (MW = 100,000 g mol^−1^) and lithium trifluoromethanesulfonate (LiCF_3_SO_3_) were purchased from Sigma-Aldrich Co. and used as received. P3HT was dissolved in 1, 2-dichlorobenzene to form a 0.5 wt% solution and stirred on a magnetic hot plate at 40 °C for 12 h. Then 3.5 μL of the P3HT solution was spin-coated on the BE at 500 rpm for 10 s, 3,000 rpm for 30 s, and then 1,500 rpm for 20 s. After spin-coating, the P3HT layer was dried on a hot plate at 100 °C for 1 h then at 142 °C for 20 min. The resulting P3HT film had a thickness of 30–50 nm. The electrolyte solution was prepared by dissolving PEO in deionized water at a concentration of 2 wt% and adding LiCF_3_SO_3_ to yield a molar ratio of 16:1 EO:Li^+^. The electrolyte solution was stirred for 12 h on a magnetic hot plate at 40 °C. The electrolyte layer was prepared by drop-casting 4 μL of the electrolyte solution on the as-prepared P3HT film, and then annealed on a hot plate at 40 or 100 °C for 20 min. A Pt top electrode (TE) with a thickness of 70 nm was deposited by the electron-beam evaporation with a rate of 0.3–0.5 Å s^−1^ through a shadow mask. A schematic of the device structure is shown in Fig. 1a.

Current–voltage (*I*–*V*) sweeps and pulse responses were measured using an Agilent B1530A waveform generator by connecting the BE and TE. For all measurements, the BE was grounded. The ambient temperature was around 26 °C and the relative humidity was about 60 during measurement. Raman spectra of the polymer films were obtained using a HR-800 Raman system. A 532 nm HeNe laser was used as the excitation source; and had a resolution of 1.32 cm^−1^.

## Results and Discussion

After the electrolyte solution was drop-cast on the as-fabricated P3HT film, the film was annealed at either 40 or 100 °C. For convenience, we named the cells made at 40 and 100 °C the low temperature cell (LTC) and high temperature cell (HTC), respectively. Figure 1b and c show the direct *I*–*V* relationship for these two cells with a voltage sweep rate of 100 V s^−1^. Conventional hysteresis loops were observed due to ion migration in the electrolyte. The residual current at *V* = 0 indicated that the ion current fell behind the voltage sweep rate. There are apparent differences between the *I*–*V* curves of the LTC and HTC. In the HTC, the absolute current was higher and the hysteresis loop was larger. Particularly, a negative differential resistance (NDR) was observed during the positive sweep for the HTC but not for the LTC (Fig. 1b). We supposed that the NDR resulted from the differences in ion mobility at the P3HT/PEO+LiCF_3_SO_3_ interface. Li^+^ ions moved through the interface under positive bias and accumulated to form an opposite electric field, which caused a current drop when the loaded bias reached the peak of the NDR. The current rise before NDR corresponded to ions doping into the P3HT layer, that during NDR corresponded to ions de-doping, and that after NDR corresponded to ions re-doping due to stronger loaded electric field. In addition, the current of the HTC was quite higher than that of LTC, suggesting that ion conductivity was higher in HTC. Both the low conductivity and absence of NDR for the LTC indicated the lack of effective or activated ions in the PEO layer.

**Fig. 2:**
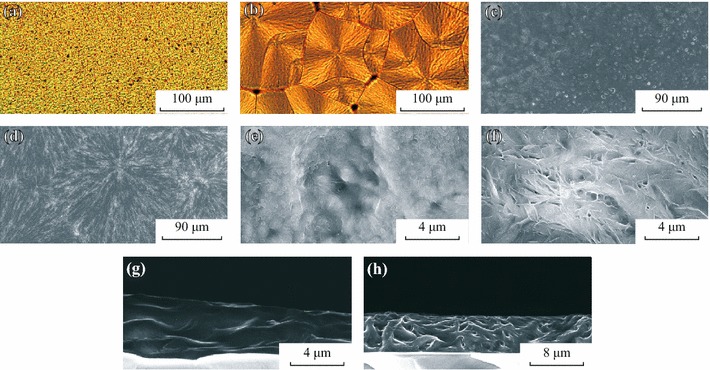
Photomicrographs and SEM images of PEO + LiCF_3_SO_3_ films annealed at different temperatures: **a**, **c**, **e**, and **g** show the films baked at 40 °C and **b**, **d**, **f**, and **h** show the films annealed at 100 °C

In order to find microstructure base accounting for the difference in Fig. 1, we examined photomicrographs and scanning electron microscope (SEM) images of the PEO+LiCF_3_SO_3_ films. Figure 2a shows that the film annealed at 40 °C presented a homogeneous surface without evident contrast. However, Fig. 2b shows that a spherulitic morphology was formed with diameters of 50–100 μm for the film annealed at 100 °C. The boundaries of the spherulitic phases were clearly observed. According to the phase diagram of PEO+LiCF_3_SO_3_, the film should be in the PEO+P(EO_3_·LiCF_3_SO_3_) phase [15]. Since the film annealed at 40 °C did not show spherulitic morphology, the LiCF_3_SO_3_ phase may be separated from the PEO phase. Finer details could be seen using SEM. Tiny particles with 0.2–0.3 μm in diameter could be observed for the 40 °C film (Fig. 2c, e), while a fibrous structure was formed during crystallization period for the 100 °C film (Fig. 2d, f). We broke the substrate in liquid nitrogen to examine the cross-sectional structure. A crosslinked fibrous structure was observed for the 100 °C film (Fig. 2h). A bundle of fiber consists of thousands of single nano-fiber, which provide nano-channels for ion migration to the P3HT layer in a defined direction.

**Fig. 3:**
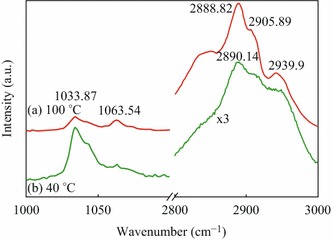
Raman spectra of the PEO + LiCF_3_SO_3_ films annealed at **a** 40 °C and **b** 100 °C

Figure 3 shows the Raman spectra of PEO+LiCF_3_SO_3_. Variations could be observed at both low wavenumbers (1,020–1,080 cm^−1^) corresponding to S=O stretching modes [16], and high wavenumbers (2888.82, 2905.89, and 2939.9 cm^−1^) corresponding to C–H stretch modes [17]. The C–H stretching modes were more evident in the 100 °C film, but were too weak to be recognized in the 40 °C film, in which the peak at 2939.9 cm^−1^ became a shoulder (Fig. 3a). The peaks at 1033.87 cm^−1^ is attributed to the S=O stretch in LiCF_3_SO_3_. The relative intensity between the C–H and S=O peaks reflects the LiCF_3_SO_3_ concentration and crystallization state of the PEO+LiCF_3_SO_3_ film. For the 40 °C film, the intensity of 1033.87 cm^−1^ was 316.904, while the intensity of 2890.14 cm^−1^ was 689.724, making *I*_(C–H)_*/I*_(S=O)_ = 2.18. For the 100 °C film, the intensity of 1033.87 cm^−1^ was 258.252 while the intensity of 2888.82 cm^−1^ was 2201.38, making *I*_(C–H)_*/I*_(S=O)_ = 8.53. This difference suggests that LiCF_3_SO_3_ was more effectively doped and dispersed into the electrolyte after annealing at 100 °C. This result was consistent with the above experiments examining the film morphologies. Combining the results obtained from the SEM and Raman experiments, we could conclude that solubility LiCF_3_SO_3_ was increased and nano-fiber structure, which is favorable for ion migration, was formed for PEO+LiCF_3_SO_3_ film annealed at 100 °C.

**Fig. 4:**
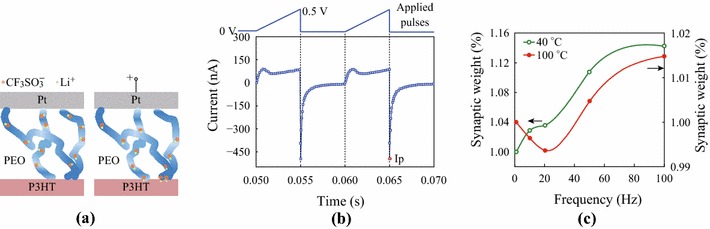
**a** Schematic of the microstructure of the pristine cell and the cell loaded by positive bias, **b** Pulse responses to a saw-tooth wave for the HTC, **c** Variation of *I*_p_ with pulse number at a frequency of 100 Hz and weight modifications of the two cells

*I*–*V* properties in Fig. 1 could be easily understood on the base of microstructure analysis. High temperature annealing enhanced the ion solubility and formed nano- channels for fast ion migration. Such channels confined ions to move in ballistic trajectory. When a positive bias was loaded, the ions moved rapidly along the nano-channels and were injected into the P3HT layer. Since counter ion was scarce in P3HT and hard to be diffused into P3HT, an inverse electric field was established quickly, and then that resulted in NDR phenomena. This process can be illustrated in Fig. 4a. The low temperature annealed PEO contained less ions so that its conductivity was very low. In addition, this sample was totally amorphous and have strong coupling with polymer segments. The loaded bias has great difficult to establish inverse field at the interface, because ions would easily find other ways out of the nano-channels to relax the system.

Therefore, a positive pulse was used as the input signal, or stimulation, to check the feasibility of realizing synaptic behavior, since frequency selectivity is useful for synaptic computation [18–20]. Results are presented in Fig. 4b, c. Pulses were applied in a shape of saw-tooth with amplitude of 0.5 V and sweep rate of 100 V s^−1^. Figure 4b shows that a single pulse response contains two parts: the current during the pulse width and the discharging current after the pulse. Since neither electrons nor holes were injected by the external field in the latter part, this process was dominated by inversely migrating ions. Therefore, the latter process could be used to mimic the ion kinetics in bio-synapses related to memory and learning. Consequently, we examined weight modifications of responses to various pulse stimulations using the same measurement paradigm as used in neuroscience [20–22]. The ratio of a response to an arbitrary frequency compared with that of the baseline frequency is the value of the weight modification. In neuroscience, the peak in the discharge process relates to the migration of ions and release of transmitter-modifying synaptic plasticity which results in learning [23]. This peak, *I*_p_ in Fig. 4b, is usually treated as either an excitatory post-synaptic current (EPSC) or inhibitory post-synaptic current (IPSC) [20–22]. Thus, we used *I*_p_ to calculate the weight modification. In addition, we used the response to smaller frequency pulses (1 Hz) as the baseline standing for the resting state of bio-synapse. However, it corresponds in here to a ‘read’ pulse with a small pulse amplitude in studies of memory and learning devices [24, 25]. The ratio of the responses to pulses with higher frequency to the baseline was calculated as the weight modification of synaptic plasticity. The pulse waveform was unchanged but the frequency was modulated during measurement to resemble an action potential in biology or neuroscience.

The values of *I*_p_ were stabilized after enough stimulation pulses. The average value of the final five responses was used to calculate the weight modification. The HTC displayed apparent frequency selectivity (Fig. 4c) similar to the spike-rate-dependent-plasticity [21, 26], i.e., it was depressed when the frequency was ranged from 1 to 20 Hz (weight < 1), but showed potentiation above 20 Hz (weight > 1). The variation in weight modification resembles the CLO learning rule first proposed to solve the selectivity problem in physiology [27, 28], where the depression at low frequencies precipitated a modest rise in [Ca^2+^] [23, 29, 30]. However, long-term plasticity was not obtained in our experiments because responses to the baseline frequency were unchanged after the cell was stimulated at frequencies higher than 1 Hz. Thus, results in Fig. 4c suggested the cell could be used as band-pass filters in synaptic computation [18–20], but not in the learning process. In addition, the LTC was not depressed at low frequencies and its weight modification monotonically increased with pulse frequency (green circle line in Fig. 4c).

Such responses were repeatable and controllable as well as that had been demonstrated in our previous study. The responses in Fig. 4b were stable during measurements. There are several controlling factors, including sweep rate, amplitude, and measurement environment influencing the responses, the weight modifications, and the threshold values. Experiments demonstrated that normal sweep rate (~100 V s^−1^) and extreme loading rate (rectangular pulse) [8] were capable of generating phenomena as shown in Fig. 4c. We did not observe depression when we used very high pulse amplitude (~2 V). The transition from depression to potentiation should be related to input energy density, in which the NDR effects should have significant influences. Systematic experiments are being designed to clarify this issue and to give the principals of selecting pulse amplitude and loading rate. By the way, the exact values of responses and weight modifications fluctuated greatly with temperature and humidity different in seasons because our studies had performed in ambient environment. Stable measurement platform should be designed to focus on another scientific issue that how the polarity molecules, such as water, facilitate, or impede the ionic kinetics at the interface.

The above transition between depression and potentiation could be explained easily using the model proposed previously [8]. Pulse frequency modulated the timing of doping, de-doping, and re-doping in a pulse width, and then modulating the amount of ions (or de-doping strength) in the discharging process after a pulse ended. If NDR effect (i.e., de-doping still existed at the end of pulse width (or window), (i.e., corresponding low frequency stimulation), depression occurred. If NDR effect finished and re-doping were launched at the end of pulse width, (i.e., corresponding high-frequency stimulation), potentiation occurred. Here, the P3HT layer acted as ion selector and fibrous PEO provided ion ballistic transportation. Thus, the cell realized frequency selectivity. This process is completely adaptive as well as ion kinetics cross membrane in bio-synapse, and the cell need not modify stimulation sign manually to obtain transition from depression to potentiation. Since the input amplitude and width are fixed, signals can be easily coded and decoded as well as that in information transport. Moreover, doping ions can be easily controlled in concentration and type, and the semiconducting polymer layer can be easily modified and doped in fabrication. Therefore, we supposed that hetero-junctions of organic semiconductor and electrolyte have extensive space in the application of information handling [18].

For the LTC samples, fibrous morphology did not form and the NDR did not appear. Therefore, there was not ionic selectivity at semiconducting polymer/electrolyte formed. Ions could diffuse laterally at the interface when strong input was loaded. Ionic kinetics was mainly dominated by behalves in electrolyte PEO. However, it had been found that no depression occurred in the pulses response of Li-doped PEO [8]. In this situation, electrical properties and pulse response resembled those in homogenous plasma system. This is the reason that the 20 Hz differentiate the depression and potentiation for HTC.

In summary, Pt/P3HT/PEO+LiCF_3_SO_3_/Pt cells were prepared and their microstructures were studied to determine the structural factors that influence the pulse responses. Nano-fibers were found in the electrolyte layer after annealed at high temperature, and the salt solubility was greatly enhanced. Ions migration could be confined in nano-channels along the fibers. This confinement was favorable and essential to form a strong inverse electric field at the P3HT/PEO+LiCF_3_SO_3_ interface during pulse width, which was related to de-doping of the semiconducting layer. Input frequency modulated timing of doping, de-doping, and re-doping to result in synaptic-like frequency selectivity, i.e., depression at low frequency stimulation and potentiation at high stimulation. Our study suggested that ions migration in direction could be facilitated to signal handling.
